# Spatiotemporal evolution, regional disparity, and driving factors of county-level rice production carbon efficiency: A case study of Jiangxi Province, China

**DOI:** 10.1371/journal.pone.0336529

**Published:** 2025-11-14

**Authors:** Beihe Wu, Jiangtao Gao, Yan Guo, Zhaojiu Chen

**Affiliations:** 1 College of Economics and Management, Jiangxi Agricultural University, Nanchang, China; 2 School of Economics and Trade, Jiangxi University of Finance and Economics, Nanchang, China; 3 Agricultural and Rural Development Research Institute, Jiangxi Academy of Social Sciences, Nanchang, China; Southwestern University of Finance and Economics, CHINA

## Abstract

Accurately quantifying the carbon efficiency of rice production (RCE) and elucidating its spatiotemporal evolution, regional disparities, and driving factors hold significant theoretical and practical implications for advancing agricultural green transformation and achieving sustainable development. Utilizing panel data from 85 counties in Jiangxi Province, China (2012–2022), this study employs a super-efficiency slack-based measure (Super-SBM) model incorporating undesirable outputs to estimate RCE. Spatial visualization via ArcGIS, kernel density estimation, Theil index decomposition, and geographical detector are applied to explore spatiotemporal patterns, regional heterogeneity, and driving mechanisms. The findings reveal that: (1) RCE exhibits a fluctuating upward trend with dynamic convergence characteristics, yet substantial improvement potential remains relative to the optimal production frontier. (2) A “central-high, peripheral-low” spatial distribution pattern dominates, accompanied by significant spatial autocorrelation and stable agglomeration features. (3) The overall Theil index initially declines before rising, with intra-regional disparities constituting the primary contributor to total differences. (4) Spatial differentiation is jointly driven by industrial and input-level factors, with distinct dominant drivers and interaction types across regions. Accordingly, we recommend formulating region-specific low-carbon policies, prioritizing key drivers, and enhancing multi-factor synergistic effects to achieve balanced regional development and facilitate agricultural green transformation.

## Introduction

Rice, as one of China’s three staple crops, exhibits production efficiency fluctuations that directly impact national food security stability [1, 2]. For a long time, the enhancement of rice production efficiency in China has predominantly relied on increased factor inputs, which has played a pivotal role in maintaining consistent yield outputs and securing food supplies [3, 4]. However, with the accelerated modernization of agriculture, the primary challenges in rice cultivation have evolved from dual coordination between resource allocation and developmental needs to a tripartite constraint system involving resource management, environmental sustainability, and production demands [5]. The conventional rice production paradigm characterized by high-input dependency and low-efficiency operation has become increasingly incongruous with contemporary agricultural requirements. This outdated approach exacerbates ecological conflicts within rice cultivation systems and poses significant threats to agricultural sustainability [6]. Empirical studies have demonstrated that current fertilizer application intensity in Chinese rice paddies reaches 338.25 kg ha^−1^, substantially exceeding the globally recommended safety threshold of 225 kg ha^−1^ [7]. Concurrently, pesticide utilization efficiency remains marginally above 40%, resulting in persistent non-point source pollution that exerts excessive pressure on arable land quality and aquatic ecosystems [8]. Furthermore, the proportion of greenhouse gas emissions from paddy fields in China accounts for 48% of total farmland emissions. Notably, methane (CH₄) emissions from paddy fields constitute 94% of this contribution, presenting substantial challenges for climate change mitigation and sustainable agricultural development [9].

Currently, China’s agricultural development has entered a critical phase of comprehensive green transformation. Confronted with multiple pressures, including tightening resource-environment constraints, escalating risks of non-point source pollution, and structural imbalances in ecosystems, there exists an urgent requirement to enhance carbon efficiency within rice production systems – achieving desired output growth while reducing redundant carbon emissions under given factor inputs [10, 11]. To attain this objective, it becomes imperative to measure carbon efficiency in rice production systems scientifically, clarify regional disparities in efficiency levels and emission reduction potentials, and identify key driving factors to accurately grasp the improvement direction and enhancement path of carbon efficiency in rice production. Therefore, the scientific quantification of carbon efficiency in rice production systems and a comprehensive analysis of its spatiotemporal evolution, regional heterogeneity, and determinant factors serve as a crucial bridge to translate macro-level carbon reduction policies into concrete green practices within agricultural operations. This endeavor addresses the pressing need to combat global climate change and achieve China’s dual-carbon goals in the agricultural sector. It also constitutes an essential strategic choice to ensure national food security while promoting environmentally sustainable agricultural development.

Carbon efficiency, serving as a critical nexus bridging economic output and carbon emissions, originated from the industrial economy domain. Its core concept postulates the theoretical minimum carbon emissions achievable under given production inputs and economic outputs, expressed as the ratio to actual emissions [12]. With intensifying resource-environment conflicts in agricultural production, carbon efficiency research has progressively extended from energy efficiency assessment to agricultural economics [13]. Existing literature predominantly examines the spatiotemporal patterns, regional disparities, and influencing factors of carbon efficiency through generalized or specialized agricultural perspectives [14–16], while carbon efficiency studies focusing on individual grain crops still have room for further exploration and deepening. As the world’s largest rice producer, China accounts for 18% of the global cultivation area and 27% of total production [17], making rice cultivation a major contributor to national agricultural carbon emissions. Although rice production generates substantial emissions, its plant growth and paddy soils exhibit significant carbon sequestration potential [18]. These dual characteristic positions carbon in rice production systems as a strategic resource, necessitating comprehensive efficiency evaluations that incorporate desirable outputs (yield and carbon sequestration) and undesirable outputs (carbon emissions).

Early studies predominantly adopted a single-factor perspective, segmenting the rice production carbon efficiency (RCE) into carbon production efficiency, economic efficiency, and ecological efficiency dimensions [3, 10, 11]. These frameworks, however, overlooked the integrated effects of production inputs and lacked systemic assessment mechanisms. Recent methodological advancements have shifted toward total-factor perspectives, primarily employing parametric and non-parametric estimation techniques. Non-parametric Data Envelope Analysis (DEA) has become prevalent in measuring carbon efficiency dynamics in rice production [19, 20]. Furthermore, to address DEA’s limitations in radial and slack adjustments, scholars have integrated Malmquist-Luenberger (ML) indices and slack-based measure (SBM) directional distance functions with DEA frameworks for analyzing efficiency evolution and regional disparities [21, 22]. Nevertheless, current indicator systems predominantly emphasize carbon sources in rice ecosystems while neglecting carbon sink valuation, resulting in incomplete systemic characterization.

Existing studies have extensively investigated the measurement methodologies, evolutionary patterns, and regional disparities of RCE, providing valuable theoretical foundations and methodological guidance for this research. However, three critical research gaps persist. First, regarding analytical perspectives, current investigations predominantly concentrate on national or regional agricultural ecosystems, while systematic investigations at the county-level scale specifically targeting rice cultivation systems remain limited. Second, methodologically, most research relies on conventional econometric models, with insufficient integration of interdisciplinary approaches combining geostatistics and econometrics. Third, in assessment frameworks, existing studies emphasize carbon emission sources within rice production systems while neglecting the carbon sequestration capacity of rice plants, resulting in incomplete evaluation systems. To address these limitations, this study establishes a refined assessment framework using panel data from 85 counties in Jiangxi Province (2012–2022). We employ a super-efficiency SBM model to quantify county-level RCE. Spatial visualization through ArcGIS, combined with Kernel density estimation, Theil index decomposition, and geographically weighted regression (GWR), enables comprehensive analysis of spatiotemporal evolution patterns, regional disparities, and driving factors of RCE.

The possible marginal contribution of this study may be as follows: First, by employing county-level panel data to analyze RCE, this research extends beyond the conventional focus on provincial or larger regional scales in existing literature, effectively addressing a critical gap. The county-scale investigation enhances understanding of spatial heterogeneity and localized driving factors, providing innovative insights for formulating targeted low-carbon agricultural policies. Second, we systematically integrated multidisciplinary methodologies, and this approach comprehensively captures both static and dynamic spatiotemporal evolution patterns of carbon efficiency while resolving slack variable issues and spatial autocorrelation concerns, thereby improving the precision of carbon efficiency evaluation. Furthermore, unlike previous studies predominantly focusing on carbon emission sources, this work establishes a comprehensive indicator system incorporating both carbon sequestration and emission processes in rice ecosystems. This dual consideration of carbon sources and sinks offers a more scientifically grounded assessment framework for achieving carbon neutrality goals in agricultural production. Collectively, these contributions strengthen the theoretical framework for agricultural carbon efficiency research while providing empirical foundations and policy references for optimizing resource allocation, mitigating regional disparities, and facilitating green transformation in rice production systems.

## Research design

### Research area and data

Jiangxi Province, situated in southeastern China (24°29′14″–30°04′43″N, 113°34′18″–118°28′56″E), occupies the southern bank of the middle-lower Yangtze River. Its topography is predominantly characterized by mountainous (36%) and hilly (42%) terrains, forming a north-opening basin surrounded by eastern, southern, and western peripheral ranges with alluvial plains in the central-northern region. Under a subtropical monsoon climate regime, the province hosts four primary grain production zones: Poyang Lake Plain, Ganfu Plain, Jitai Basin, and western Jiangxi region. As one of China’s 13 major grain-producing provinces, Jiangxi holds a national high typical and representative nationwide in rice cultivation, consistently ranking third in paddy output and playing a pivotal role in safeguarding national food security [23]. However, with the accelerated advancement of agricultural modernization, the per-unit-area inputs of agricultural production materials (e.g., energy, chemical fertilizers, and pesticides) in Jiangxi Province’s grain production system have shown a persistent upward trend since 2012. This trajectory not only diminishes local agricultural productivity but also substantially increases greenhouse gas emissions [24]. Consequently, while rice production in Jiangxi Province significantly contributes to national food security, it concurrently emerges as a significant source of regional agricultural carbon emissions, imposing considerable pressure on local agro-ecosystems. Furthermore, the elevated carbon emission levels indicate that Jiangxi’s rice production systems face intensified challenges in achieving the “dual carbon” goals within the agricultural sector, necessitating urgent exploration of effective emission mitigation and carbon sequestration strategies.

### Research methodology

#### Measuring RCE with super-SBM model.

The conventional DEA model neglected slack variables in input-output processes, failed to effectively differentiate decision-making units (DMUs) with efficiency scores equal to or greater than 1, and compromised the accuracy of efficiency evaluations [25]. To address these limitations, this study adopted a super-efficiency slacks-based measure (SBM) model incorporating non-desirable outputs under constant returns to scale (CRS), building on established methodologies [26]. The specific formulation for measuring RCE is expressed as follows:


$$\rho =min\frac{\text{1}+\frac{\text{1}}{m}\sum_{i=\text{1}}^{m}\frac{\overset{―}{x}}{x_{ik}}}{\text{1}-\frac{\text{1}}{r_{\text{1}}+r_{\text{2}}}(\sum_{s=\text{1}}^{r_{\text{1}}}\frac{{\overset{―}{y}}^{d}}{{\overset{―}{y}}_{sk}^{d}}+\sum_{q=\text{1}}^{r_{\text{2}}}\frac{{\overset{―}{y}}^{u}}{{\overset{―}{y}}_{qk}^{u}})}$$ (1)

$$s.t.\left\{ \begin{array}{c} \overset{―}{x}\geq \sum_{j=\text{1},\neq k}^{n}x_{ij}\lambda_{j};{\overset{―}{y}}^{d}\leq \sum_{j=\text{1},\neq k}^{n}y_{sj}^{d}\lambda_{j}\\ {\overset{―}{y}}^{u}\geq \sum_{j=\text{1},\neq k}^{n}y_{qj}^{u}\lambda_{j};\overset{―}{x}\geq x_{k};{\overset{―}{y}}^{d}\leq {\overset{―}{y}}_{k}^{d};{\overset{―}{y}}^{u}\geq {\overset{―}{y}}_{k}^{u}\\ \lambda_{j}\geq \text{0};i=\text{1},\text{2},\cdots ,m;j=\text{1},\text{2},\cdots ,n\\ s=\text{1},\text{2},\cdots ,r_{\text{1}};q=\text{1},\text{2},\cdots ,r_{\text{2}} \end{array}\right.\;$$ (2)

In the model, *ρ* represents the REE of counties in Jiangxi Province, values ≥1 indicate optimal frontier performance, and *n* denotes the number of DMUs, corresponding to 85 county-level units in Jiangxi. Each DMU incorporates *m* inputs, *r*_*1*_ desirable outputs, and *r*_*2*_ undesirable outputs. The slack variables $\overset{―}{x}$, ${\overset{―}{y}}^{d}$, and ${\overset{―}{y}}^{u}$ represent the amounts of excess input, deficit in desirable output, and excess in undesirable output, respectively. For instance, in the context of RCE, $\overset{―}{x}$ could indicate redundant fertilizer usage, while ${\overset{―}{y}}^{d}$signifies the portion of carbon emissions that could be reduced without compromising output. $x_{ij}$, $y_{sj}^{d}$, and $y_{qj}^{u}$ denote the optimized input *i*, desirable output *s*, and undesirable output *q* for DMU *j* after slack adjustment. $\lambda_{j}$ denotes the weight coefficient.

#### Kernel density estimate.

To investigate the dynamic evolutionary trends of RCE across Jiangxi Province and its grain production functional zones, this study employed kernel density estimation [27]. The equation is expressed as follows:


$$\overset{´}{f}(D)=\frac{\text{1}}{nh}\sum_{i=\text{1}}^{n}K\left(\frac{\overset{―}{D}-D_{i}}{h}\right)$$ (3)

In the above formula, $D_{i}$ is the RCE in *i* county, $\overset{―}{D}$ is the mean value of the RCE of all counties, n is the number of counties, h is the bandwidth, *k*(•) is a kernel function, which is defined as a Gaussian kernel function in this study.

#### Spatial autocorrelation analysis.

Spatial autocorrelation analysis revealed spatial association and heterogeneity phenomena in geographic data [28]. The global spatial autocorrelation approach examined the overall spatial association degree across the region by computing the global Moran’s I index, Z-score, and p-value [29]. This study utilized global and local spatial autocorrelation methods to investigate the spatial association degree and spatial heterogeneity characteristics of RCE, respectively. The formula is expressed as follows:


$$Moran^{\prime }s\;I=\frac{\sum_{i=\text{1}}^{n}\sum_{j=\text{1}}^{n}w_{ij}(x_{i}-\overset{―}{x})(x_{j}-\overset{―}{x})}{S^{\text{2}}\sum_{i=\text{1}}^{n}\sum_{j=\text{1}}^{n}w_{ij}}$$ (4)

In equation 4, I denote the global Moran′s I index, n represents the number of counties, $x_{i}$ and $y_{j}$ correspond to RCE in counties *i* and *j*, respectively. $\overset{―}{x}$ and $S^{\text{2}}$ denote the sample mean and variance, $w_{ij}$ is the spatial weight matrix. The global Moran′s I statistic ranges between −1 and 1. Values I > 0 indicate positive spatial autocorrelation (clustered patterns), I < 0 suggest negative spatial autocorrelation (dispersed patterns), and I = 0 implies a random spatial distribution with no significant correlation.

The global Moran’s I index is primarily employed to examine overall spatial differences, yet it may overlook heterogeneous characteristics within local regions [30]. In contrast, the LISA (Local Indicators of Spatial Association) statistic derived from the local Moran’s I index serves as a localized measure of spatial autocorrelation, enabling the analysis of spatial clustering patterns within specific sub-regions [31]. The formulas are expressed as follows:


$${LISA}_{i}=\frac{x_{i}-\overset{―}{x}}{S^{\text{2}}}\sum_{j}^{n}\left[w_{ij}(x_{i}-\overset{―}{x})\right]$$ (5)

In equation 5, ${LISA}_{i}$ represents the local correlation coefficient for county *i*. ${LISA}_{i}$>0 indicates spatial positive correlation between county *i* and its adjacent counties regarding carbon efficiency, where the spatial clusters in the region can be categorized as either high-high (H-H) or low-low (L-L) agglomerations. Conversely, ${LISA}_{i}$<0 suggests a spatial negative correlation, with the corresponding spatial clusters classified as either high-low (H-L) or low-high (L-H) agglomerations.

#### Theil index.

The Theil index is capable of decomposing overall regional disparities into inter-regional and intra-regional differences, thereby enabling the identification of primary sources of disparities and their respective contribution rates [32]. Accordingly, this study employed the Theil index to assess regional disparities in RCE across Jiangxi Province. The specific equations are formulated as follows:


$$T=T_{w}+T_{b}=\sum_{k=\text{1}}^{k}y_{k}T_{k}+\sum_{k=\text{1}}^{k}y_{k}ln\left(\frac{y_{k}}{\frac{n_{k}}{n}}\right)$$

$$T_{k}=\sum_{{i\in g}_{k}}\frac{y_{i}}{y_{k}}ln\left(\frac{\frac{y_{i}}{y_{k}}}{\frac{\text{1}}{k_{n}}}\right)$$ (6)

In the above equation, T denotes the overall regional disparity, while $T_{w}$ and $T_{b}$ represent within-region and between-region disparities, respectively. $T_{k}$ indicates the within-group disparity for the k-th group, where *k* is the number of groups, each denoted as $g_{k}\left(k=\text{1},∙∙∙,k\right)$. $n_{k}$ represents the number of individuals in the k-th group, with the constraint that $\sum_{k=\text{1}}^{k}n_{k}=n$. $y_{i}$ and $y_{k}$ correspond to carbon efficiency at the primary partitioning level (group classification) and secondary partitioning level (individual units), respectively.

Furthermore, the contribution rates of within-group, intraregional, and interregional disparities can be calculated based on the above equations:

$\text{within}-\text{group}:\;D_{k}=y_{k}\frac{T_{k}}{T}$


$$\text{intraregional}:\;D_{w}=\sum_{k=\text{1}}^{k}\frac{D_{k}}{T}$$

$$\text{interregional}:\;D_{b}=\frac{T_{b}}{T}$$ (7)

#### Geographic detector.

Geographic detector is a statistical method designed to analyze specific attributes’ spatial heterogeneity and examine their driving factors’ explanatory power [33]. In this study, the factor detector within Geodetector identifies the degree of influence of driving factors on the spatial divergence of carbon efficiency. In contrast, the interaction detector effectively evaluates how combined effects between different drivers modify their collective explanatory power on carbon efficiency values [34]. The factor detector is formulated as follows:


$$q=\text{1}-\frac{\sum_{h=\text{1}}^{L}n_{h}\sigma_{h}^{\text{2}}}{n\sigma^{\text{2}}}$$ (8)

In equation 8, q denotes the explanatory power of each factor on the spatial differentiation of RCE, where q ∈ [0, 1]. A higher q value indicates stronger explanatory power. L represents the stratification of each factor; n and $n_{h}$ denote the sample sizes of the entire study area and the h-th stratum, respectively; ${\omega \text{t}\sigma }^{\text{2}}$ and ${\omega \text{t}\sigma }_{h}^{\text{2}}$ correspond to the variances of the entire study area and the h-th stratum, respectively.

To further evaluate the interactive effects among driving factors, we compared interaction values with independent q values, thereby categorizing inter-factor interactions into five types (Table 1).

**Table 1:** Interaction types and criteria for driving factors.

Interaction Relationship	Type
q (M∩N)> Max (q (M), q (N))	Bivariate enhancement
q (M∩N) <Min (q (M), q (N))	Nonlinear weakening
q (M∩N) = q (M) + q (N)	Independent interaction
q (M∩N) > q (M) + q (N)	Nonlinear enhancement
Min (q (M), q (N)) < q (M∩N) <Max (q (M), q (N))	Single-factor nonlinear weakening

It should be noted that the q-value quantifies the extent to which the spatial heterogeneity of driving factors explains the spatial heterogeneity of the dependent variable. Although no significance testing is performed for the q-value, it retains a clear physical interpretation and does not rely on assumptions of normal distribution [35]. Furthermore, when applying the geographical detector to identify drivers of spatial heterogeneity for a given phenomenon, all continuous driving variables must be discretized into categorical variables. Accordingly, this study first categorized all driving factors using the K-means clustering method in SPSS software. The geographical detector was then utilized to quantify the influence of each factor on the spatial heterogeneity of RCE. It is noteworthy that the optimal number of clusters for the K-means clustering analysis of all factors in the study was determined using the elbow method, a widely adopted approach that balances within-cluster variance and model simplicity to enhance the statistical robustness of the classification in the geographical detector analysis.

### Indicator selection and construction

#### Construction of index system for RCE.

Drawing on existing research findings [36–38], this study establishes an input indicator system comprising three dimensions: labor, land, and agricultural factor inputs. Rice yield and carbon sequestration are considered as desirable outputs, while carbon emissions are treated as the undesirable output. The specific indicators and their corresponding descriptions are detailed in Table 2.

**Table 2:** Input‒output indicator system for RCE assessment.

Category	Variable	Explanation	Units
Input	Labor	Labor force engaged in rice production	10⁴ persons
	Land	Cultivated area for rice	10³ hectares
	Chemical fertilizer	Fertilizer consumption in rice production	10⁴ metric tons
	Pesticide	Pesticide application in rice production	10⁴ metric tons
	Agricultural film	Plastic film utilization in rice production	10⁴ metric tons
	Agricultural diesel	Diesel fuel consumption in rice production	10⁴ metric tons
Desirable Output	Economic benefit	Rice yield	10⁴ metric tons
	Ecological benefit	Rice ecosystem carbon sink	10⁴ metric tons CO₂-eq
Undesirable Output	Environmental cost	Total carbon emissions from rice farming	10⁴ metric tons CO₂-eq

(1) Input indicators. To ensure consistency between input indicators and rice output metrics, this study employs a weighting coefficient method [39] to disaggregate production factors. Two coefficients are defined: A = (Agricultural Output Value/ Total Output Value of Agriculture, Forestry, Animal Husbandry, and Fishery) × (Rice Sown Area/ Total Sown Area of Crops); B = Rice Sown Area/ Total Sown Area of Crops. Land input retains the rice sown area metric. Labor input is calculated as Coefficient A × Agricultural, forestry, animal husbandry, and fishery industry employees, and all other input indicators are multiplied by Coefficient B.
(2) Output Indicators. Carbon sink estimation. Rice carbon sinks are quantified following the methodology proposed by Chen et al. [40]:

$$C_{s}=C_{r}\times Y_{r}\times (\text{1}-W_{r})\times (\text{1}+R_{r})/H_{r}$$ (9)

In equation 9, *C*_*s*_ denotes the total carbon sink of rice cultivation, *C*_*r*_ represents the photosynthetic carbon assimilation rate of rice, *Y*_*r*_ is the economic yield of rice crops, *W*_*r*_ is the moisture content of the economic product, *R*_*r*_ is the root-to-shoot ratio coefficient, and *H*_*r*_ is the harvest index (economic coefficient). The rice crops’ carbon absorption rate, water content, economic coefficient, and root-shoot ratio were set to 0.41,0.12,0.45 and 0.60, respectively. These parameters are empirically derived from physiological studies and field measurements of commonly cultivated rice varieties in the study region, with supporting references duly cited.

Calculation of carbon emissions from rice cultivation. Total carbon emissions from rice production systems encompass direct and indirect carbon emission equivalents generated throughout the entire cultivation cycle, from field tillage to harvest. Drawing on established methodologies [18, 41, 42], this study quantifies carbon emissions using the emission factor approach, addressing two primary sources: agricultural input utilization and rice growth processes. To account for data limitations in tillage area quantification, the actual sown area of rice is adopted as a proxy for tillage area. All other agricultural inputs are multiplied by coefficient B. The proposed model is formulated as follows:


$$C_{e}=\sum_{k=\text{1}}^{n}e_{k}=\sum_{k=\text{1}}^{n}\delta_{k}\cdot \omega$$ (10)

In equation 10, *C*_*e*_ denotes the total carbon emissions from the rice production system (kg CO₂-eq), where *k* represents emission source categories (*k = 1,2,3,…*), encompassing agricultural inputs and rice growth processes. The *e*_*k*_ quantifies emissions from each source, with *δ* and *ω* corresponding to the emission factor and actual input quantity of the source, respectively. For analytical consistency, methane (CH₄) emissions are converted to carbon equivalents using the standardized conversion: 1 t CH₄ = 6.82 t CO₂-eq. Emission factors for all sources and their referenced literature are detailed in Table 3.

**Table 3:** Carbon-emission sources and coefficients.

Category	Emission Source	Coefficient	Unit	Source
Agricultural Inputs	Nitrogen fertilizer	1.74	kg CO₂-eq kg ⁻ ¹	[40]
	Phosphorus fertilizer	0.20		
	Potassium fertilizer	0.15		
	Compound fertilizer	0.38		[43]
	Chemical pesticides	4.9341		[44]
		Agricultural film	5.18	
	Agricultural diesel	0.5927		[45]
	Irrigation	20.476	kg CO₂-eq ha ⁻ ¹	[46]
	Tillage	312.6	kg CO₂-eq km ⁻ ²	[47]
Rice Growth	Early-season rice	154.70	kg CH₄ ha ⁻ ¹	[48]
	Mid-season rice	654.20		
	Late-season rice	458.00		

Note: The emission factors presented in the table were derived from authoritative, peer-reviewed studies focused on the Chinese agricultural system, thereby ensuring their applicability and representativeness within the regional context of Jiangxi Province.

#### Selection of driving factors for RCE.

Existing studies indicate that the natural environment, economic development, and social characteristics are three primary factors influencing agricultural production efficiency [49]. Given the unique characteristics of RCE, this study constructs a driver factor indicator system from three dimensions: agricultural industry development, production input factors, and socio-environmental features (Table 4).

**Table 4:** Indicator system for driving factors of RCE.

Category	Driving Factor	Measurement	Unit
Industrial	Agricultural productive service level (X1)	Output value of agricultural productive services/ Gross agricultural output	–
	Agricultural industrial structure (X2)	Grain output value/ Total agricultural output	–
	Operation scale (X3)	Rice cultivation area/ Labor input in rice production	ha/person
Input	Chemical input intensity (X4)	Total chemical input in rice production/ Rice cultivation area	t/ha
	Mechanization level (X5)	Total agricultural machinery power/ Cultivated area	kW/ha
	Agricultural consumption rate (X6)	Total agricultural consumption/ Gross agricultural output	–
Social	Urbanization rate (X7)	Urban population/ Total population	%
	Financial support for agriculture (X8)	Expenditure on agriculture, forestry, and water affairs/ Local fiscal budget	–
	Per capita GDP (X9)	Regional GDP/ Total population	CNY/person

For the agricultural industry development dimension, and based on data availability, we selected agricultural productive service level, agricultural industrial structure, and rice operation scale as indicators. In the agricultural input dimension, prior research has demonstrated that production input factors and resource consumption levels directly affect agricultural green total factor productivity [50]. Accordingly, this study adopts chemical input intensity, mechanization level, and agricultural consumption rate as indicators. Regarding socio-environmental features, urbanization level, fiscal support for agriculture, and per capita GDP have been shown to optimize the allocation of capital, labor, and production technologies, thereby influencing agricultural production carbon efficiency [49, 51]. Therefore, this study selected urbanization rate, financial support for agriculture, and per capita GDP as socio-environmental indicators.

## Results and analysis

### Temporal evolution of RCE

#### Static analysis.

Based on the previously established input-output indicator system for RCE, this study measures and analyzes the temporal evolution patterns of county-level RCE in Jiangxi Province from 2012 to 2022 using a Super-SBM model. As presented in Table 5, the results focus on the annual mean values for each region rather than displaying efficiency values for all individual counties; detailed temporal data are available upon request.

**Table 5:** RCE in counties of Jiangxi Province from 2012 to 2022.

Zones	All counties	Main grain producing zones				Non-major grain producing zones
Year		Poyang Lake Plain	Ganfu Plain	Jitai Basin	Western Jiangxi Province	
2012	0.4459	0.4519	0.5010	0.4310	0.5520	0.3803
2013	0.4698	0.5099	0.5343	0.4441	0.5628	0.3823
2014	0.4724	0.4831	0.5351	0.4754	0.5690	0.3829
2015	0.4739	0.4769	0.5323	0.4723	0.5859	0.3953
2016	0.4888	0.4931	0.5485	0.4922	0.4916	0.4229
2017	0.4804	0.4883	0.5623	0.4735	0.5041	0.3946
2018	0.4853	0.4673	0.5711	0.4962	0.5305	0.3951
2019	0.5137	0.4982	0.6232	0.5113	0.5915	0.4035
2020	0.5607	0.5396	0.6888	0.5370	0.6241	0.4557
2021	0.6078	0.6030	0.7462	0.5736	0.6534	0.4931
2022	0.6676	0.6467	0.7982	0.6477	0.7500	0.5536
Mean	0.5151	0.5144	0.6037	0.5050	0.5832	0.4236

Firstly, the analysis of all the counties in Jiangxi Province revealed that the mean value of RCE demonstrated a consistent annual increase from 0.4459 in 2012 to 0.6676 in 2022, representing a 49.72% improvement over the decade. These findings indicate substantial progress in low-carbon transformation within Jiangxi’s rice industry during 2012–2022. Specifically, under equivalent production input conditions, the system achieved enhanced economic and ecological benefits while maintaining carbon emission constraints. Alternatively, equivalent output levels could be sustained with reduced carbon emissions. This improvement can be attributed to two primary factors: (1) accelerated agricultural modernization with improved infrastructure and enhanced production services, and (2) policy-driven reduction in chemical inputs, effectively curbing emission sources. Nevertheless, current carbon efficiency levels remain 33.24% below optimal production frontiers, suggesting significant potential for further emission reduction through technological optimization and input management.

Secondly, the regional analysis demonstrated distinct spatial variations in carbon efficiency across Jiangxi Province during the study period. Provincial averages for carbon efficiency reached 0.5151, with grain-producing counties (0.5491) substantially outperforming non-major grain-producing counties (0.4236). Among core production regions, the Ganfu Plain core production area achieved the highest efficiency (0.6037), followed by the Western Jiangxi high-yield belt (0.5832), Poyang Lake Plain core area (0.5144), and Jitai Basin core area (0.5050). All production regions exceeded non-grain county averages. The superior performance in the Ganfu Plain stemmed from favorable natural conditions combined with mechanization and scale-intensive operations. Conversely, non-major grain-producing counties exhibited persistently low-efficiency levels due to environmental constraints and lagging adoption of green production technologies. While all regions showed measurable efficiency gains during the study period, substantial inter-regional disparities persist, necessitating tailored emission reduction strategies based on regional characteristics and emission sources.

#### Dynamic analysis.

**Fig 1:**
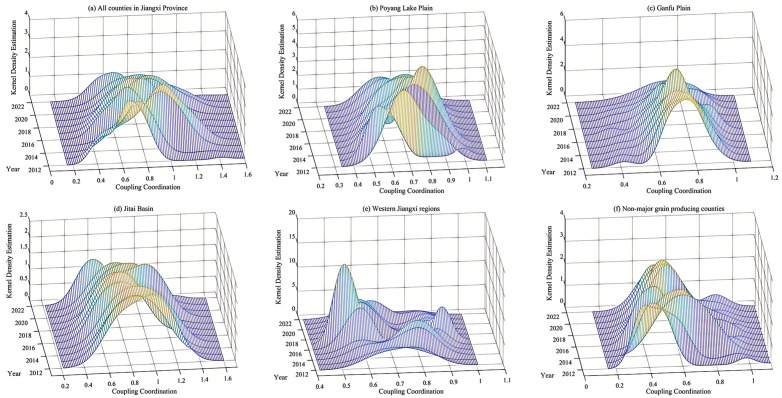
Dynamic convergence of RCE.

To facilitate comparison, this study employed the kernel density estimation method to analyze the dynamic evolution trends of RCE across Jiangxi Province and its major grain functional regions (Fig 1).

Firstly, analysis from all counties in Jiangxi Province (Fig 1(a)) shows that the RCE curve exhibited a rightward shift from 2012 to 2022, accompanied by a significant expansion in bandwidth and right-tailed characteristics. The vertical height of the main peak decreased substantially while the peak value of the side peak gradually increased. These observations indicate an overall upward trend in carbon efficiency across the province, albeit with reduced spatial clustering and the emergence of a multipolar evolution pattern. Compared to 2012, the 2015 curve showed a slight increase in vertical height, a minor rightward shift of the main peak, and narrowed bandwidth, suggesting initial dynamic convergence characteristics. By 2018, the vertical height decreased, the main peak shifted leftward, and bandwidth expanded, reflecting declining efficiency and dynamic divergence. In 2022, the curve displayed a pronounced decline in vertical height, leftward peak shift, expanded bandwidth with right-tailed features, and rising side peak values. This implies convergent characteristics in carbon efficiency but widening regional disparities, where certain counties exhibited efficiency levels exceeding the provincial average, significantly elevating overall performance.

Secondly, analyze different main grain-producing areas. In the Poyang Lake Plain region (Fig 1(b)), the carbon efficiency curve shifted rightward overall, with alternating fluctuations in main peak height. Compared to 2012, the 2015 curve showed a significant increase in main peak height, while subsequent years exhibited declining peak values alongside increasingly evident right-tailed and bandwidth expansion trends. This demonstrates rising carbon efficiency coupled with reduced spatial clustering and growing inter-county disparities. The Ganfu Plain region (Fig 1(c)) displayed gradual rightward shifts of the efficiency curve during 2012–2022, with sustained decreases in main peak height, progressive bandwidth expansion, and left-tailed characteristics. These patterns suggest improved overall efficiency but declining spatial cohesion, where subpar performance in some counties constrained regional progress. Contrastingly, the Jitai Basin region (Fig 1(d)) exhibited leftward curve shifts accompanied by significant bandwidth expansion and right-tailed features, indicating dynamic divergence characteristics. Growing regional disparities emerged, with several counties surpassing regional averages and driving overall efficiency improvements. The Western Jiangxi high-yield zone (Fig 1(e)), comprising only four counties (Xiangdong, Lianhua, Shangli, and Luxi), showed no clear evolutionary trend but demonstrated progressive improvements in carbon efficiency levels. For non-core grain production regions (Fig 1(f)), while curve positions remained stable across observation periods, the main peak height displayed fluctuating declines alongside increasing bandwidth expansion and right-tailed characteristics. This reflects dynamic divergence patterns with expanding regional disparities despite maintaining an overall upward efficiency trajectory.

In core grain-producing areas, the convergence of efficiency levels among regions has slowed, with certain regions showing marked divergence from the central trend. This divergence is particularly evident in regions with distinct agro-ecological conditions, where resource endowments and production practices differ significantly from neighboring areas. The widening gap between high-efficiency and low-efficiency regions suggests that spatial spillover effects are becoming less pronounced, potentially due to localized technological lock-in or policy implementation variations. Meanwhile, in non-core production regions, the persistence of multi-peak distributions indicates persistent structural heterogeneity, with some regions lagging due to limited access to advanced agricultural technologies or constrained by topographical factors. These patterns collectively underscore the need for region-specific policy interventions rather than uniform efficiency-enhancement strategies across different grain production zones. Furthermore, the observed multipolar evolution and dynamic convergence underscore the growing spatial heterogeneity in carbon efficiency within rice production systems, with potential driving factors to be examined in detail in subsequent chapters.

### Spatial evolution and agglomeration characteristics of RCE

#### Spatial evolution characteristics of RCE.

To investigate the spatial distribution characteristics and evolutionary patterns of carbon efficiency in rice production across counties in Jiangxi Province, this study evaluated carbon efficiency in 85 county-level administrative units in 2012, 2015, 2018, and 2022. Owing to space constraints, the detailed carbon efficiency values for all counties in the selected years are provided in S1 Table 1 of the Supplementary Material at the end of this article.

Overall, the RCE in Jiangxi’s counties exhibited an upward trend from 2012 to 2022, characterized by a spatial and evolutionary pattern of “high central and low peripheral” At the observed time points (2012, 2015, 2018, and 2022), the mean carbon efficiency values were 0.4459, 0.4739, 0.4853, and 0.6676, respectively, indicating accelerated growth in later years. Spatially, high-efficiency zones in 2012 were relatively clustered, centred on the Ganfu Plain core production area and gradually diminishing toward peripheral regions, with overall efficiency remaining low. By 2022, these high-efficiency zones transitioned to a scattered distribution. Although the overall efficiency improved, spatial disparities intensified due to widening regional gaps. Geographically, carbon efficiency displayed pronounced spatial heterogeneity. High-efficiency areas predominantly occurred in flat terrains such as the Ganfu Plain and Poyang Lake Plain core production zones. In contrast, low-efficiency areas clustered in southern regions of the Jitai Basin core production area and hilly non-core production counties in southern Jiangxi. The hierarchical pattern remained stable: Ganfu Plain > western Jiangxi > Poyang Lake Plain > Jitai Basin > non-major grain-producing counties. Notably, while all counties achieved varying degrees of improvement in carbon efficiency, spatial inequity persisted without significant mitigation. Despite some counties reaching optimal efficiency frontiers, carbon emission redundancy remained prevalent in most regions, posing potential bottlenecks for agricultural green transition.

#### Spatial agglomeration characteristics of RCE.

**Fig 2:**
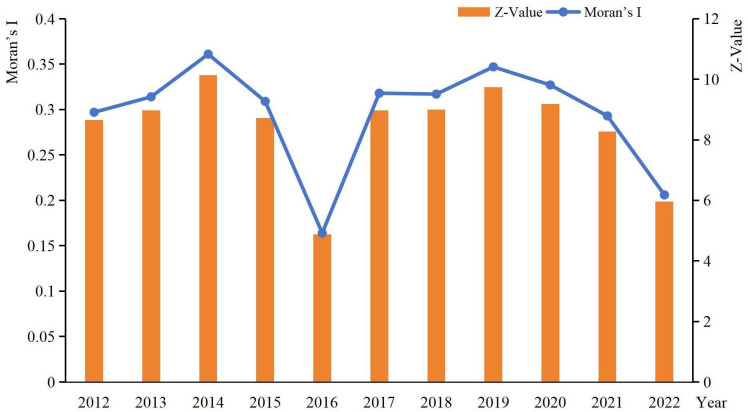
Global Moran’s I of RCE.

This study commenced by constructing a spatial weight matrix and then calculated the Global Moran’s I of RCE using Stata software (Fig 2). The Moran’s I values from 2012 to 2022 consistently passed the Z-test at the 1% significance level, with most coefficients ranging between 0.2 and 0.4. These results demonstrate significant positive spatial autocorrelation in RCE across county-level regions in Jiangxi Province, indicating a relatively stable spatial clustering pattern throughout the study period.

To further elucidate the spatial agglomeration patterns of carbon efficiency in rice production, this study calculated the local Moran’s I index for rice production carbon efficiency across 85 counties in Jiangxi Province for the years 2012, 2015, 2018, and 2022. The resulting LISA (Local Indicators of Spatial Association) cluster maps clearly illustrate the spatial clustering characteristics (see Supplementary Material S1 Table 2 at the end of the article). Based on the spatial agglomeration patterns, the regions were categorized into four types: high–high (H–H), low–low (L–L), high–low (H–L), and low–high (L–H).

First is the analysis of H-H agglomeration zones. The spatial distribution of H-H agglomeration zones exhibited a transition from contiguous to scattered patterns over the study period. Initially concentrated in the northern Jitai Basin, the core gradually shifted to the central Ganfu Plain. In 2012, 12 counties—including Qingyuan District, Ji’an County, Jishui County, Anfu County, and Yongxin County—formed the H-H agglomeration zone, demonstrating notable spatial clustering. By 2015, seven additional counties (e.g., Nanchang County, Jinxian County, Xiangdong District, Xiajiang County, Fengcheng City, Zhangshu City, and Gao’an City) were incorporated, with a prominent high-yield cluster emerging in western Jiangxi, diversifying the agglomeration pattern. In 2018, three more counties (Fengcheng City, Xinjian District, and Wanzai County) expanded the H-H cluster to 17 counties, predominantly in the northern Jitai Basin and central Ganfu Plain, reflecting their superior carbon efficiency in rice production. By 2022, the number of H-H counties decreased to 11, displaying a fragmented distribution with core clusters concentrated around Nanchang City. This dynamic suggests that the central Ganfu Plain achieved significant progress in agricultural green transformation, where enhanced factor mobility, technology diffusion, and spillover effects improved carbon efficiency in rice production. These advancements elevated local performance and drove regional low-carbon development by radiating benefits to neighbouring counties.

Secondly, analyze the L-L agglomeration zones. The L-L agglomeration zones showed an initial decline followed by an increase in county numbers, primarily distributed in the southern Jitai Basin and non-grain-producing counties of the hilly southern Jiangxi region. In 2012, 13 counties—including Ganxian District, Xinfeng County, Dayu County, Chongyi County, and Anyuan County—constituted the L-L cluster. By 2015, the number decreased to 11 counties (excluding Dayu and Chongyi). The L-L cluster remained stable in 2018 but expanded to 14 counties by 2022, including the Nankang District. This persistence highlights the slow green transition in southern Jiangxi’s non-grain-producing counties, constrained by natural environmental limitations and lagging production technologies, which maintained low carbon efficiency in rice production and stabilized the spatial scope of L-L agglomerations.

Finally, the H-L and L-H agglomeration zones are analyzed. Between 2012 and 2022, these zones involved a limited number of counties with geographically dispersed distributions. These zones demonstrated high sensitivity to the spatial dynamics of neighbouring counties, reflecting localized spillover effects or efficiency disparities.

### Spatial difference analysis of RCE

**Fig 3:**
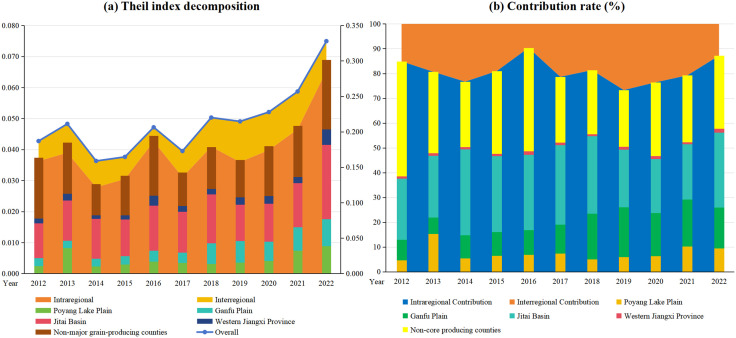
Theil index decomposition and its contribution rate.

To investigate the regional disparities in RCE across Jiangxi Province and the emission-reducing potential, this study employed the Theil index to analyze spatial differences in carbon efficiency (Fig 3). The results indicated that the overall Theil index of RCE in Jiangxi Province exhibited a trend of initial decline followed by an increase during 2012–2022, reflecting a dynamic shift from early-stage convergence to later-stage divergence in regional disparities. This shift may be attributed to the gradual equalization of agricultural infrastructure development and industrial progress across regions in the early phase, which fostered coordinated improvements in carbon efficiency and thereby reduced regional disparities. However, divergences in natural conditions, production technologies, and pathways for low-carbon agricultural development emerged over time. Additionally, certain counties accelerated their agricultural green transition by leveraging locational advantages, collectively driving the observed divergence in carbon efficiency. The interregional Theil index remained relatively stable, while the intraregional Theil index aligned with the overall provincial trend. Contribution rate analysis revealed that intraregional disparities accounted for 70%–90% of the total Theil index throughout the study period. Given the distinct policy frameworks governing each functional area, the primary reason regional internal disparities constitute the main source of overall variation may lie in the significant differences across functional areas with respect to agricultural development priorities, utilization of resource endowments, and the extent of policy implementation. For example, functional areas primarily oriented toward grain production tend to prioritize increasing rice yields, resulting in relatively limited adoption and diffusion of carbon reduction technologies. In contrast, ecological conservation-oriented functional areas actively promote green agricultural practices and enhance investment in low-carbon production technologies to safeguard environmental quality. These divergent strategies lead to substantial variations in rice production carbon efficiency across functional areas, thereby rendering intra-regional differences the dominant component of overall disparities.

Furthermore, analysis of the Theil index differences in carbon efficiency and their contribution rates within Jiangxi Province’s grain functional zones from 2012 to 2022 revealed the following findings. The mean contribution rates of the Theil index were ordered as follows: non-major grain-producing counties > Jitai Basin > Ganfu Plain > Poyang Lake Plain > Western Jiangxi. Notably, the contribution rate of non-major grain-producing counties exhibited a declining trend, while the main grain-producing zones showed an upward trend. This indicates that regional disparities in carbon efficiency among non-major grain-producing counties are narrowing, whereas differences among counties in grain-producing zones are widening. Specifically, from 2012 to 2016, non-major grain-producing counties consistently maintained the highest Theil index contribution rate, followed by the Jitai Basin, while Western Jiangxi remained the lowest. From 2017 to 2022, however, the contribution rate of the Jitai Basin surpassed that of non-major grain-producing counties. These results suggest that the Jitai Basin primarily drives regional disparities in RCE within Jiangxi Province, whereas Western Jiangxi has sustained a relatively balanced. The observed patterns can be attributed to variations in natural conditions, agricultural policies, and technological advancements across regions. Non-major grain-producing counties, characterized by diversified agricultural structures, may face limitations in promoting and applying agricultural technologies, leading to more significant carbon efficiency disparities. In contrast, grain-producing zones, particularly the Poyang Lake Plain and Ganfu Plain, benefit from concentrated rice cultivation and advanced agricultural technologies, resulting in smaller carbon efficiency disparities. Despite its extensive rice cultivation, the increasing disparity in the Jitai Basin production area may correlate with recent adjustments in agricultural policies. Meanwhile, Western Jiangxi demonstrates minimal carbon efficiency differences due to its balanced agricultural production conditions and uniform adoption of technological practices.

### Analysis of driving factors on RCE

#### Single factor detection.

To investigate the influence of driving factors on the spatial differentiation of RCE, this study first applied K-means clustering via SPSS software to categorize all driving factors. Subsequently, geodetector analysis was employed to perform single-factor detection on each influencing factor across the entire study period (Table 6). The results demonstrated that all nine indicators selected from three dimensions — industrial development, factor input, and social environment — exhibited statistical significance at the 1% level, though with varying q-values. This indicates that the identified factors collectively exerted significant driving effects on the RCE yet displayed distinct explanatory capacities.

**Table 6:** Detection results of driving factors for RCE.

Variable	X1	X2	X3	X4	X5	X6	X7	X8	X9
q-value	0.0682	0.1304	0.2118	0.2908	0.0405	0.0711	0.1689	0.0358	0.1272
p-value	0.0000	0.0000	0.0000	0.0000	0.0000	0.0000	0.0000	0.0716	0.0000

**Fig 4:**
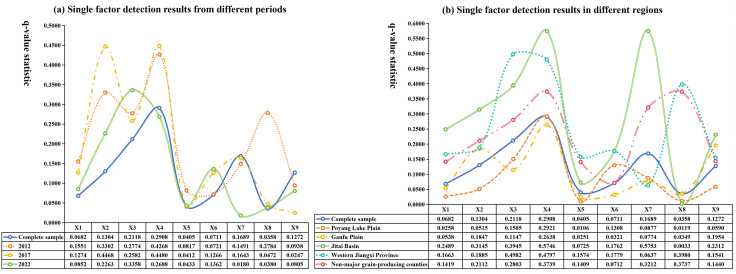
Single factor detection results for different periods and regions.

Accordingly, to systematically compare differences in these explanatory capacities, individual factor detection was conducted across multiple sample periods and regional contexts (Fig 4). Furthermore, considering manuscript constraints, the top four factors ranked by q-value were identified as primary drivers for detailed interpretation. In contrast, secondary driving factors were excluded from subsequent causal analysis to maintain conciseness.

First, the analysis of single-factor detection results across different periods (Fig 4(a)) reveals that chemical input intensity, operation scale, and agricultural industrial structure were consistently identified as key factors influencing RCE throughout all study periods. The elevated chemical input intensity is closely linked to increased fertilizer and pesticide usage, which, while enhancing rice yields, concurrently contributes to higher carbon emissions. The expansion of operation scale generally improves production efficiency and optimizes resource utilization, thereby positively influencing carbon efficiency. Meanwhile, optimizing agricultural industrial structure enhances land-use efficiency and reduces reliance on chemical inputs, effectively lowering carbon emissions. These findings suggest that rational regulation of chemical inputs, scientifically planned operation scaling, and structural adjustments in agricultural industries could significantly improve carbon efficiency and promote sustainable rice production.

Second, regional single-factor detection results (Fig 4(b)) demonstrate significant spatial heterogeneity in dominant drivers: In the Poyang Lake Plain, the primary drivers were chemical input intensity (q = 0.2921), operation scale (0.1505), agricultural consumption rate (0.1308), and urbanization rate (0.0877). The Ganfu Plain exhibited key drivers of chemical input intensity (0.2638), per capita GDP (0.1954), agricultural industrial structure (0.1847), and operation scale (0.1147). For the Jitai Basin, urbanization rate (0.5753), chemical input intensity (0.5746), operation scale (0.3945), and agricultural industrial structure (0.3145) dominated. The Western Jiangxi showed operation scale (0.4982), chemical input intensity (0.4797), financial support for agriculture (0.3980), and agricultural industrial structure (0.1885) as primary factors. In non-major grain-producing counties, chemical input intensity (0.3739), financial support for agriculture (0.3737), urbanization rate (0.3212), and operation scale (0.2803) were critical. Notably, chemical input intensity emerged as a universal determinant across all regions, underscoring the necessity of reducing fertilizer and pesticide application for emission mitigation. Spatial variations highlight the need for region-specific strategies, including optimized operation scale planning, rational urbanization management, and agricultural structural adjustments. Therefore, the above factors should be considered, and corresponding measures should be taken to promote the sustainable development of rice production when formulating regional agricultural development strategies.

#### Factor interactive detection.

To further evaluate the interaction effects among driving factors, this study employed Origin 2022 software to generate heatmaps that visually present the factor interaction detection results of RCE across different sampling periods and regions.

**Fig 5:**
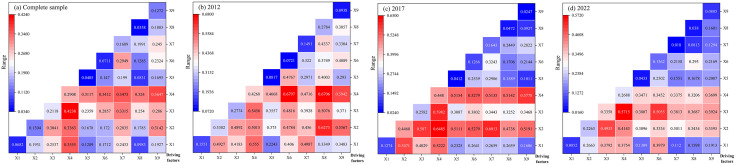
Factor interaction detection results in different periods.

First, the interaction detection results of factors across different periods were analyzed (Fig 5). The results indicated that all diagonal values in the matrix were lower than those in their respective rows and columns, implying that the interactive effects of any two driving factors on RCE exceeded the impact of individual factors. As summarized in Table 1, the interaction types were broadly categorized as bifactorial enhancement and nonlinear enhancement. Specifically, during the whole sample period, the interaction between operation scale (∩) chemical input intensity exhibited the most decisive influence, with a q-value of 0.4238. In 2012, the interaction between chemical input intensity (∩) agricultural consumption rate dominated (q = 0.6797), while in 2017, the interaction between agricultural industrial structure (∩) chemical input intensity showed the highest impact (q = 0.6485). By 2022, the interaction between operation scale (∩) chemical input intensity regained prominence (q = 0.5715). Overall, the spatial heterogeneity of RCE was primarily driven by synergistic effects between industrial- and factor-level determinants, with bifactorial enhancement being the predominant interaction type. Social-level factors mainly exerted foundational constraints and support on carbon efficiency, while their spatial heterogeneity was profoundly influenced by interactions between industrial- and factor-level factors. Consequently, enhancing the RCE requires coordinated optimization of industrial-level adjustments and factor-level improvements.

**Fig 6:**
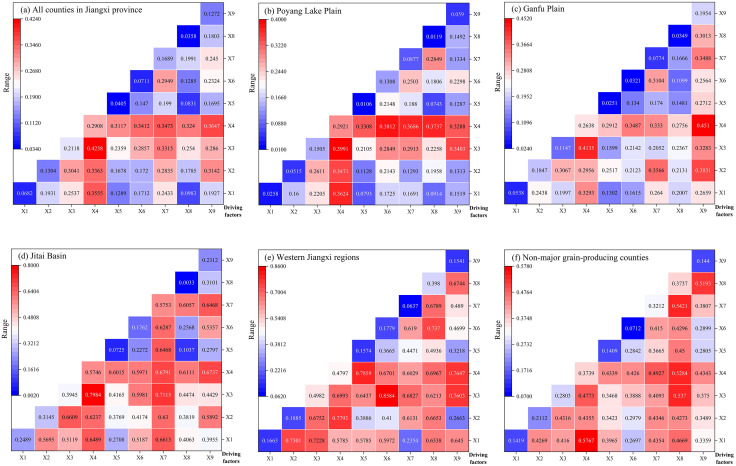
Factor interaction detection results in different regions.

Second, regional-specific interaction detection results were examined (Fig 6). In the Poyang Lake Plain, chemical input intensity demonstrated strong interactive effects with other factors, predominantly manifesting as bifactorial enhancement. In contrast, the Ganfu Plain exhibited more significant influence from interactions involving per capita GDP, with nonlinear enhancement being the dominant interaction pattern. The Jitai Basin showed significant interactive effects between chemical input intensity and other factors, again dominated by bifactorial enhancement. Notably, in the Western Jiangxi and non-major grain-producing counties, the interactive effects of any two factors consistently surpassed individual factor impacts, often exceeding the sum of individual effects, with nonlinear enhancement prevailing.

The aforementioned results underscore the importance of formulating targeted agricultural policies and measures tailored to the specific conditions of different grain functional zones. In the Poyang Lake Plain, a key grain-producing region, priority should be given to optimizing the use of chemical inputs and enhancing their synergistic integration with other agricultural practices to improve carbon efficiency. In the Ganfu Plain, another major production area, policy efforts should focus on balancing the growth of per capita GDP with sustainable agricultural development, thereby achieving co-benefits for economic progress and environmental protection. In the Jitai Basin, where agricultural output is significant, coordinated optimization of chemical input levels and urbanization rates is essential to mitigate potential adverse effects on carbon efficiency. For high-yield regions in western Jiangxi and non-core grain-producing counties, policymakers are advised to consider the cumulative impacts of multiple socioeconomic and ecological factors and to advance the green transformation of agriculture through innovations in technology and management practices.

## Discussion

The findings of this study provide critical insights into the spatiotemporal evolution, regional disparities, and driving factors of RCE at the county scale in Jiangxi Province. These results align with existing literature on agricultural carbon efficiency but also reveal unique spatial and temporal dynamics specific to rice-dominated agroecosystems.

Firstly, in terms of research methods. Applying the non-radial super-efficiency SBM model effectively addresses slack variables and non-desirable outputs, offering a more nuanced efficiency assessment than traditional DEA approaches. However, excluding climate variables and market fluctuations may limit the model’s ability to capture exogenous shocks [52]. Future studies could incorporate dynamic environmental variables or machine learning techniques to enhance predictive accuracy. Additionally, while the geodetector method successfully identified key drivers, its reliance on spatial autocorrelation assumes static relationships over time. Longitudinal analyses using panel data models could better disentangle causality and temporal lags in driving factors.

Secondly, regarding the spatiotemporal dynamics of RCE and its regional differences. The upward trend in RCE over the study period (2012–2022) reflects the cumulative effects of agricultural modernization and policy interventions, such as promoting green production technologies and reducing chemical inputs. Similar trends have been observed in other rice-producing regions, such as the Mekong Delta and India’s Punjab, where mechanization and precision farming contributed to efficiency gains while curbing emissions [52, 53]. However, the persistence of significant regional disparities—particularly the “high-central, low-peripheral” spatial pattern—suggests that uneven resource allocation and heterogeneous adoption of sustainable practices remain critical barriers. This echoes findings in China’s Yangtze River Basin, where core agricultural zones often exhibit higher efficiency due to better infrastructure and economies of scale [54]. The dynamic convergence of RCE highlights the role of technological spillovers and policy diffusion. However, the widening gap between high- and low-efficiency counties underscores the need for targeted interventions in lagging regions.

Thirdly, the regional heterogeneity in RCE calls for spatially differentiated strategies. In high-efficiency zones (e.g., Ganfu Plain), policies should prioritize maintaining technological leadership through precision agriculture and carbon trading mechanisms; for lagging regions (e.g., southern Jiangxi), targeted investments in rural infrastructure, farmer training, and low-carbon subsidies are critical.

Finally, comparative analysis of the spatiotemporal heterogeneity of drivers. The dominance of chemical input intensity (X4) as a key driver across all regions resonates with global studies emphasizing the dual role of fertilizers and pesticides in enhancing yields while exacerbating emissions [55]. Notably, the interactive effects between chemical input intensity and operation scale (X3) or agricultural industrial structure (X2) suggest that optimizing input-use efficiency through scale management and crop diversification could yield synergistic benefits. For instance, larger farms in the Ganfu Plain likely benefit from advanced irrigation systems and integrated pest management, reducing per-unit emissions—a phenomenon documented in U.S. Midwest corn production [56]. Conversely, the weaker explanatory power of socioeconomic factors like urbanization rate (X7) and financial support for agriculture (X8) implies that broader developmental policies may indirectly or delay impacts on RCE, necessitating tighter integration of environmental goals into regional planning frameworks.

Furthermore, our research reveals distinct configurations of driving factors across different regions. For instance, in the Jitai Basin, the urbanization rate emerges as the dominant factor, whereas in the Ganfu Plain, per capita GDP plays a predominant role. These findings underscore the importance of avoiding uniform policy approaches and instead adopting region-specific strategies. The methodological framework and localized insights presented in this study offer valuable references for formulating targeted low-carbon development policies in other major rice-producing regions in China—such as Hunan and Anhui—that share similar agro-ecological and socio-economic characteristics.

While this study provides new perspectives and methods for investigating the spatiotemporal dynamics and drivers of RCE, several potential limitations should be acknowledged. First, this study’s reliance on county-level aggregated data may mask intra-county variations in farming practices. Future research could employ micro-level surveys or remote sensing data to refine spatial resolution. Furthermore, the carbon sequestration potential of rice paddies was estimated using fixed coefficients, which may not fully account for soil heterogeneity or microbial dynamics. Incorporating field-measured carbon fluxes would strengthen the robustness of RCE assessments. Lastly, expanding the scope to include downstream supply chain emissions could provide a more holistic view of rice production’s carbon footprint.

## Conclusions and recommendations

Based on the panel data of 85 counties in Jiangxi Province from 2012 to 2022, this paper calculates RCE using the SBM model. It analyzes the spatial and temporal evolution, regional differences, and driving factors of RCE in Jiangxi Province through ArcGIS, kernel density estimation, Theil index, and geographical detector. The following conclusions are drawn:

(1) Temporal evolution characteristics: During 2012–2022, the RCE in Jiangxi Province exhibited a fluctuating upward trend, yet significant potential for improvement remains compared to the optimal production frontier. Regional disparities were pronounced, with distinct evolutionary trajectories across regions. For instance, in 2022, under current input and technological conditions, redundant carbon emissions from rice production could theoretically be reduced by 33.24%, indicating substantial emission reduction potential. Furthermore, while dynamic convergence was observed in carbon efficiency across the province, regional disparities expanded, reflecting a multipolar evolution pattern. Certain counties with efficiency levels above the provincial average played a pivotal role in elevating overall efficiency.
(2) Spatial patterns and evolution: The RCE displayed a “central high, peripheral low” spatial distribution characterized by significant positive spatial correlation and stable agglomeration effects. High-high and low-low agglomeration clusters followed specific distribution patterns and evolutionary rules. In contrast, high-low and low-high clusters exhibited dispersed distributions, rendering them more susceptible to fluctuations in neighboring regions.
(3) Regional disparities: The Theil index of RCE across the province of Jiangxi initially declined and then increased, revealing a shift from convergence to divergence in regional disparities. Intra-regional differences constituted the primary source of overall disparity. The mean contribution rates of Theil indices across functional grain production zones ranked as follows: non-major grain-producing counties > Jitai Basin > Ganfu Plain > Poyang Lake Plain > western Jiangxi.
(4) Driving factors: Spatial differentiation in carbon efficiency was jointly driven by industrial and factor-level determinants, with dominant drivers and interaction effects varying regionally. Social-level factors primarily imposed foundational constraints or provided structural support, while the interactive effects of industrial and factor-level variables predominantly shaped spatial heterogeneity.

Based on the above conclusions, to improve the RCE in county-level areas of Jiangxi Province and achieve sustainable agricultural development, the following suggestions are proposed:

First, develop regionally differentiated low-carbon policies: Tailored strategies should align with local conditions. The Ganfu Plain and Poyang Lake Plain core production areas should optimize agricultural structures and chemical inputs to consolidate efficiency gains. The Jitai Basin area requires balanced optimization of chemical inputs and urbanization rates to mitigate intra-regional disparities. The western Jiangxi high-yield zone should sustain balanced development through improved agricultural conditions and technology adoption. At the same time, non-grain-producing counties should prioritize multifactor synergies to accelerate the green transition.

Second, it is essential to strengthen the development of agricultural infrastructure and promote balanced regional growth within the rice industry. Priority should be given to enhancing the role of the Poyang Lake Plain—a key grain-producing region—as a leader in carbon-efficient rice production. This strategic focus will drive the advancement of precision agriculture technologies, expand access to green mechanized services, and improve carbon efficiency in surrounding counties. Such efforts will not only elevate the overall level of agricultural green development in the region but also gradually optimize the spatial distribution of carbon efficiency in rice production, thereby mitigating the widening of regional disparities.

Third, target key drivers and synergistic effects: Implement region-specific interventions based on dominant driving factors while accounting for interaction effects. Coordinated optimization of industrial and factor-level variables should be prioritized to reduce carbon redundancy, enhance efficiency, and advance low-carbon, high-efficiency rice production systems, thereby accelerating Jiangxi’s agricultural green transition.

## Supporting information

S1 Table Spatiotemporal Evolution of Carbon Use Efficiency in Rice Production Across Counties of Jiangxi Province. (DOCX)

S1 Table Spatial LISA Agglomeration Patterns of Carbon Efficiency in Rice Production Across Counties of Jiangxi Province. (DOCX)

S1 Text Data. (XLS)
