# Supplementary material for: Spatiotemporal evolution, regional disparity, and driving factors of county-level rice production carbon efficiency: A case study of Jiangxi Province, China
Source: PLoS One. 2025 Nov 14;20(11):e0336529. doi: 10.1371/journal.pone.0336529 (PMC12617899; doi:10.1371/journal.pone.0336529)
Supplement: S1 Table — (DOCX) [file pone.0336529.s001.docx]

**S1** **Table 1. Spatiotemporal Evolution of Carbon Use Efficiency in Rice Production Across Counties of Jiangxi Province.**

| ID | County name | 2012 | 2015 | 2018 | 2022 |
| --- | --- | --- | --- | --- | --- |
| 1 | Xinjian | 0.5185 | 0.5850 | 0.6621 | 1.0912 |
| 2 | Nanchang | 0.5405 | 0.6207 | 0.6769 | 0.8960 |
| 3 | Anyi | 0.4455 | 0.4835 | 0.5532 | 0.6907 |
| 4 | Jinxian | 0.5367 | 0.5982 | 0.6408 | 0.6743 |
| 5 | Fuliang | 0.4118 | 0.4519 | 0.3906 | 0.4656 |
| 6 | Leping | 0.4675 | 0.5011 | 0.4512 | 0.5283 |
| 7 | Xiangdong | 0.5235 | 0.5848 | 0.5242 | 0.6424 |
| 8 | Lianhua | 0.6235 | 0.6275 | 0.6424 | 1.0054 |
| 9 | Shangli | 0.4602 | 0.4842 | 0.4708 | 0.5934 |
| 10 | Luxi | 0.6008 | 0.6470 | 0.4848 | 0.7586 |
| 11 | Chaisang | 0.4244 | 0.4226 | 0.4306 | 1.0812 |
| 12 | Wuning | 0.4251 | 0.3378 | 0.3769 | 0.4133 |
| 13 | Xiushui | 0.3399 | 0.3410 | 0.3499 | 0.3969 |
| 14 | Yongxiu | 0.4203 | 0.4303 | 0.3798 | 0.5703 |
| 15 | De'an | 0.4657 | 0.4731 | 0.4088 | 0.5082 |
| 16 | Duchang | 0.5495 | 0.5692 | 0.4268 | 1.1135 |
| 17 | Hukou | 0.3791 | 0.4298 | 0.3770 | 0.6492 |
| 18 | Pengze | 0.2781 | 0.3185 | 0.2746 | 0.3766 |
| 19 | Ruichang | 0.3479 | 0.3850 | 0.3968 | 0.5095 |
| 20 | Lushan | 0.4394 | 0.4823 | 0.3720 | 0.4599 |
| 21 | Yushui | 0.4752 | 0.4480 | 0.4327 | 0.5257 |
| 22 | Fenyi | 0.4454 | 0.4367 | 0.4735 | 0.6274 |
| 23 | Yujiang | 0.5911 | 0.5863 | 0.6166 | 0.9235 |
| 24 | Guixi | 0.3942 | 0.4891 | 0.5270 | 0.7551 |
| 25 | Nankang | 0.4274 | 0.4706 | 0.4419 | 0.4825 |
| 26 | Ganxian | 0.2533 | 0.2529 | 0.2451 | 0.3137 |
| 27 | Xinfeng | 0.2544 | 0.2909 | 0.2842 | 0.3427 |
| 28 | Dayu | 0.2736 | 0.2974 | 0.3195 | 0.3574 |
| 29 | Shangyou | 0.3230 | 0.3475 | 0.3655 | 0.4335 |
| 30 | Chongyi | 0.2879 | 0.7123 | 0.3245 | 0.3710 |
| 31 | Anyuan | 0.2181 | 0.1601 | 0.1542 | 0.1882 |
| 32 | Longnan | 0.2805 | 0.3075 | 0.2976 | 0.3366 |
| 33 | Dingnan | 0.2734 | 0.2702 | 0.2678 | 0.3848 |
| 34 | Quannan | 0.2017 | 0.2209 | 0.3098 | 0.3636 |
| 35 | Ningdu | 0.3087 | 0.3472 | 0.3223 | 0.4248 |
| 36 | Yudu | 0.2219 | 0.2468 | 0.2528 | 0.3199 |
| 37 | Xingguo | 0.3419 | 0.3660 | 0.3441 | 0.4006 |
| 38 | Huichang | 0.2343 | 0.2758 | 0.3088 | 0.3813 |
| 39 | Xunwu | 0.2011 | 0.2408 | 0.2192 | 0.2882 |
| 40 | Shicheng | 0.2366 | 0.2655 | 0.2820 | 0.3294 |
| 41 | Ruijin | 0.3437 | 0.3601 | 0.3903 | 0.4549 |
| 42 | Qingyuan | 0.6446 | 0.6902 | 0.6875 | 0.7155 |
| 43 | Ji'an | 0.5675 | 0.6955 | 0.6397 | 0.6463 |
| 44 | Jishui | 0.5673 | 0.6355 | 0.6881 | 1.6003 |
| 45 | Xiajiang | 0.5587 | 0.5974 | 0.6054 | 0.6301 |
| 46 | Xin'gan | 0.4447 | 0.5000 | 0.5440 | 1.0038 |
| 47 | Yongfeng | 0.5203 | 0.5259 | 0.5267 | 0.5740 |
| 48 | Taihe | 0.4745 | 0.4785 | 0.4741 | 1.0020 |
| 49 | Suichuan | 0.4012 | 0.4370 | 0.4925 | 0.6244 |
| 50 | Wan'an | 0.4758 | 0.4769 | 0.5120 | 0.5853 |
| 51 | Anfu | 0.5543 | 0.6478 | 0.6594 | 0.7998 |
| 52 | Yongxin | 0.5951 | 0.6777 | 1.0090 | 1.0051 |
| 53 | Jingangshan | 1.0670 | 0.6773 | 0.8235 | 1.0482 |
| 54 | Yuanzhou | 0.5730 | 0.6454 | 0.7913 | 1.0198 |
| 55 | Fengxin | 0.5953 | 0.5433 | 0.5967 | 0.6943 |
| 56 | Wanzai | 0.5112 | 0.5475 | 0.6069 | 0.8122 |
| 57 | Shanggao | 0.5563 | 0.5722 | 0.7630 | 1.0083 |
| 58 | Yifeng | 0.5794 | 0.5402 | 0.5540 | 0.6707 |
| 59 | Jing'an | 0.5207 | 0.5380 | 0.5814 | 1.0082 |
| 60 | Tonggu | 0.3088 | 0.3350 | 0.4301 | 0.6274 |
| 61 | Fengcheng | 0.5260 | 0.5724 | 0.6349 | 0.8246 |
| 62 | Zhangshu | 0.5290 | 0.6299 | 0.7120 | 1.0265 |
| 63 | Gaoan | 0.5265 | 0.6064 | 0.7239 | 1.0082 |
| 64 | Linchuan | 0.4527 | 0.4747 | 0.3416 | 0.4236 |
| 65 | Dongxiang | 0.4547 | 0.4914 | 0.5461 | 1.0219 |
| 66 | Nancheng | 0.6403 | 0.6642 | 0.7236 | 1.0290 |
| 67 | Lichuan | 0.4330 | 0.4315 | 0.5067 | 1.0330 |
| 68 | Nanfeng | 0.2693 | 0.3130 | 0.2460 | 0.3115 |
| 69 | Chongren | 0.5323 | 0.5179 | 0.5489 | 0.8614 |
| 70 | Le'an | 0.4782 | 0.5097 | 0.4840 | 0.7958 |
| 71 | Yihuang | 0.4690 | 0.5091 | 0.4801 | 0.7345 |
| 72 | Jinxi | 0.4354 | 0.5025 | 0.4373 | 0.5783 |
| 73 | Zixi | 0.5257 | 0.4703 | 0.4870 | 0.7065 |
| 74 | Guangchang | 0.4316 | 0.3729 | 0.4399 | 0.5818 |
| 75 | Guangfeng | 0.4884 | 0.5378 | 0.5142 | 0.6635 |
| 76 | Guangxin | 0.4506 | 0.4983 | 0.5258 | 0.7904 |
| 77 | Yushan | 0.5038 | 0.5445 | 0.5475 | 0.7131 |
| 78 | Yanshan | 0.3788 | 0.3538 | 0.3647 | 0.4863 |
| 79 | Hengfeng | 0.4304 | 0.4597 | 0.6139 | 1.0219 |
| 80 | Geyang | 0.3915 | 0.4641 | 0.4561 | 0.5736 |
| 81 | Yugan | 0.4427 | 0.5087 | 0.4846 | 0.5782 |
| 82 | Poyang | 0.4360 | 0.5107 | 0.5658 | 0.7321 |
| 83 | Wannian | 0.5385 | 0.5063 | 0.4995 | 0.6432 |
| 84 | Wuyuan | 0.4109 | 0.4486 | 0.4508 | 0.5365 |
| 85 | Dexing | 0.4221 | 0.4572 | 0.4603 | 0.5644 |
