# Supplementary material for: Spatiotemporal evolution, regional disparity, and driving factors of county-level rice production carbon efficiency: A case study of Jiangxi Province, China
Source: PLoS One. 2025 Nov 14;20(11):e0336529. doi: 10.1371/journal.pone.0336529 (PMC12617899; doi:10.1371/journal.pone.0336529)
Supplement: S1 Table — (DOCX) [file pone.0336529.s002.docx]

**S1** **Table 2. Spatial LISA Agglomeration Patterns of Carbon Efficiency in Rice Production Across Counties of Jiangxi Province.**

| ID | County name | 2012 | 2015 | 2018 | 2022 |
| --- | --- | --- | --- | --- | --- |
| 1 | Xinjian | Not significant | Not significant | H-H | H-H |
| 2 | Nanchang | Not significant | H-H | H-H | H-H |
| 3 | Anyi | Not significant | Not significant | Not significant | Not significant |
| 4 | Jinxian | Not significant | H-H | H-H | Not significant |
| 5 | Fuliang | Not significant | Not significant | Not significant | Not significant |
| 6 | Leping | Not significant | Not significant | Not significant | Not significant |
| 7 | Xiangdong | Not significant | H-H | Not significant | Not significant |
| 8 | Lianhua | H-H | H-H | H-H | H-H |
| 9 | Shangli | Not significant | Not significant | Not significant | Not significant |
| 10 | Luxi | H-H | H-H | Not significant | L-L |
| 11 | Chaisang | Not significant | Not significant | Not significant | Not significant |
| 12 | Wuning | Not significant | Not significant | Not significant | Not significant |
| 13 | Xiushui | Not significant | Not significant | Not significant | Not significant |
| 14 | Yongxiu | L-L | Not significant | Not significant | Not significant |
| 15 | De'an | L-L | Not significant | Not significant | Not significant |
| 16 | Duchang | Not significant | Not significant | Not significant | Not significant |
| 17 | Hukou | Not significant | Not significant | Not significant | Not significant |
| 18 | Pengze | Not significant | Not significant | Not significant | Not significant |
| 19 | Ruichang | Not significant | Not significant | Not significant | Not significant |
| 20 | Lushan | Not significant | Not significant | Not significant | Not significant |
| 21 | Yushui | Not significant | Not significant | Not significant | Not significant |
| 22 | Fenyi | Not significant | Not significant | Not significant | Not significant |
| 23 | Yujiang | Not significant | Not significant | Not significant | Not significant |
| 24 | Guixi | Not significant | Not significant | Not significant | Not significant |
| 25 | Nankang | Not significant | Not significant | Not significant | L-H |
| 26 | Ganxian | L-H | L-H | L-H | L-H |
| 27 | Xinfeng | L-H | L-H | L-H | L-H |
| 28 | Dayu | L-H | Not significant | L-H | L-H |
| 29 | Shangyou | Not significant | Not significant | L-L | Not significant |
| 30 | Chongyi | L-H | L-L | L-H | L-H |
| 31 | Anyuan | L-H | L-H | L-H | L-H |
| 32 | Longnan | L-H | L-H | L-H | L-H |
| 33 | Dingnan | L-H | L-H | L-H | L-H |
| 34 | Quannan | L-H | L-H | L-H | L-H |
| 35 | Ningdu | Not significant | Not significant | Not significant | Not significant |
| 36 | Yudu | L-H | L-H | L-H | L-H |
| 37 | Xingguo | Not significant | Not significant | Not significant | Not significant |
| 38 | Huichang | L-H | L-H | L-H | L-H |
| 39 | Xunwu | L-H | L-H | L-H | L-H |
| 40 | Shicheng | L-H | L-H | L-H | L-H |
| 41 | Ruijin | L-H | L-H | L-H | L-H |
| 42 | Qingyuan | H-H | H-H | H-H | Not significant |
| 43 | Ji'an | H-H | H-H | H-H | Not significant |
| 44 | Jishui | H-H | H-H | H-H | H-H |
| 45 | Xiajiang | Not significant | H-H | H-H | Not significant |
| 46 | Xin'gan | Not significant | Not significant | Not significant | H-H |
| 47 | Yongfeng | Not significant | Not significant | Not significant | Not significant |
| 48 | Taihe | Not significant | Not significant | Not significant | Not significant |
| 49 | Suichuan | Not significant | Not significant | Not significant | Not significant |
| 50 | Wan'an | Not significant | Not significant | Not significant | Not significant |
| 51 | Anfu | H-H | H-H | H-H | Not significant |
| 52 | Yongxin | H-H | H-H | H-H | H-H |
| 53 | Jingangshan | H-H | H-H | H-H | H-H |
| 54 | Yuanzhou | H-H | H-H | H-H | Not significant |
| 55 | Fengxin | H-H | Not significant | Not significant | Not significant |
| 56 | Wanzai | Not significant | Not significant | H-H | Not significant |
| 57 | Shanggao | H-H | Not significant | H-H | Not significant |
| 58 | Yifeng | H-H | Not significant | Not significant | Not significant |
| 59 | Jing'an | Not significant | Not significant | Not significant | Not significant |
| 60 | Tonggu | H-L | H-L | Not significant | Not significant |
| 61 | Fengcheng | Not significant | H-H | H-H | H-H |
| 62 | Zhangshu | Not significant | H-H | H-H | H-H |
| 63 | Gaoan | Not significant | H-H | H-H | H-H |
| 64 | Linchuan | Not significant | Not significant | H-L | H-L |
| 65 | Dongxiang | Not significant | Not significant | Not significant | H-H |
| 66 | Nancheng | Not significant | Not significant | Not significant | Not significant |
| 67 | Lichuan | Not significant | Not significant | Not significant | Not significant |
| 68 | Nanfeng | H-L | Not significant | H-L | H-L |
| 69 | Chongren | Not significant | Not significant | Not significant | Not significant |
| 70 | Le'an | Not significant | Not significant | Not significant | Not significant |
| 71 | Yihuang | Not significant | Not significant | Not significant | Not significant |
| 72 | Jinxi | Not significant | Not significant | Not significant | Not significant |
| 73 | Zixi | Not significant | Not significant | Not significant | Not significant |
| 74 | Guangchang | Not significant | Not significant | Not significant | Not significant |
| 75 | Guangfeng | Not significant | Not significant | Not significant | Not significant |
| 76 | Guangxin | Not significant | Not significant | Not significant | Not significant |
| 77 | Yushan | Not significant | Not significant | Not significant | Not significant |
| 78 | Yanshan | Not significant | Not significant | Not significant | L-L |
| 79 | Hengfeng | Not significant | Not significant | Not significant | Not significant |
| 80 | Geyang | Not significant | Not significant | Not significant | Not significant |
| 81 | Yugan | Not significant | Not significant | Not significant | Not significant |
| 82 | Poyang | Not significant | Not significant | Not significant | Not significant |
| 83 | Wannian | Not significant | Not significant | Not significant | Not significant |
| 84 | Wuyuan | Not significant | Not significant | Not significant | Not significant |
| 85 | Dexing | Not significant | Not significant | Not significant | Not significant |
